# Editorial: Targeted Therapy for Pituitary Adenomas

**DOI:** 10.3389/fendo.2019.00358

**Published:** 2019-06-04

**Authors:** Takako Araki, Odelia Cooper, Hidenori Fukuoka

**Affiliations:** ^1^Division of Diabetes, Endocrinology and Metabolism, Department of Medicine, University of Minnesota, Minneapolis, MN, United States; ^2^Pituitary Center, Cedars-Sinai Medical Center, Los Angeles, CA, United States; ^3^Division of Diabetes and Endocrinology, Kobe University Hospital, Kobe, Japan

**Keywords:** pituitary tumors, targeted therapeutic, aggressive pituitary tumor, Cushing's disease, Somatostatin agonists, Dopamin agonists, molecular targeted agents

Although pituitary tumors are mostly benign, patients often face impaired quality of life and increased morbidity and mortality secondary to hormone hypersecretion, mass effect symptoms, and the multiple therapies used for the underlying tumor. While surgical resection is the mainstay of treatment for the majority of these tumors, medical therapy may be used preoperatively for hormone and mass stabilization as well as for treatment of residual/and recurrent tumors. Targeted, precision-based therapies for pituitary tumors have been developed and are increasingly used effectively for these situations. In the current research topic in “Frontiers in Endocrinology,” we present studies and reviews on approved and investigative targeted therapies for pituitary tumors.

Pituitary tumors are derived from lineage specific hormone secreting pituitary trophic cells, namely lactotroph, somatotroph, corticotroph, thyrotroph, and gonadotroph, and are associated with hormone specific clinical features. Lactotroph tumors present with hyperprolactinemia and symptoms of galactorrhea and hypogonadism. Somatotroph tumors manifest growth hormone (GH) hyper-secretion and present with acral changes, soft tissue swelling, impaired metabolism, and respiratory and cardiac diseases. Clinical features of corticotroph tumors with adrenocortiocotropic (ACTH) hyper-secretion include central obesity, metabolic abnormalities, osteoporosis, immunosuppression, and psychiatric disorders. Thyrotroph tumors can result in hyperthyroidism. Gonadotroph tumors rarely show excessive secretion of intact luteinizing hormone (LH) or follicle stimulating hormone (FSH) and are therefore considered non-functioning adenomas (NFAs). The goal of medical therapy for pituitary tumors is to normalize hormone secretion in functioning tumors to alleviate hormone specific clinical features as well as to control tumor growth in both functioning and non-functioning tumors.

Somatostatin receptor (SSTR) ligands (SRLs) are the first-line therapy for somatotroph tumors as they reduce GH secretion and tumor size. Recently, pasireotide, a second generation SRL targeting SSTR 1, 2, 4, and 5, has been approved for acromegaly. In addition to these drugs, novel SRLs are currently being developed, including DG3173 (SRL target to SSTR 2, 4, and 5), CAM2029 (subcutaneous depot SRL), VP-003 (subcutaneous implants SRL), and oral SRLs. These therapies are reviewed by Paragliola and Salvatori.

Dopamine agonists which target the dopamine subtype 2 receptor (D2R) are the primary therapy for prolactinomas. As somatotroph, corticotroph, and non-functioning tumors express D2R, dopamine agonists are a potential avenue of therapy for these masses, and the evidence for their use is reviewed by Cooper and Greenman.

Aggressive pituitary tumors and carcinomas are at high risk of recurrence and are resistant to standard therapies. Temozolomide (TMZ), an alkylating agent which is used to treat gliomas, has been shown to be effective in these challenging pituitary tumors. The mechanism of action of TMZ is mediated by DNA methylation, which results in cell death during the sequence of mismatch repair. Syro et al. discuss the role of TMZ for aggressive tumors, and its future directions.

Pituitary apoplexy can occur in pituitary tumors due to intra-tumoral hemorrhage or infarction. Risk factors for apoplexy include anticoagulant drugs, hormone stimulation testing, intra-cranial surgery, or dopamine agonists. However, the pathogenesis of pituitary apoplexy is largely unknown. In this issue, Gupta and Dutta discuss the putative molecular mechanisms of this disease, including the role of VEGF, TNF-α, pituitary tumor-transforming gene (PTTG), matrix metalloproteinase-2/9 (MMP-2/9), and hypoxia-inducing factor (HIF). These insights may lead to future development of novel biomarkers and targeted therapy for pituitary apoplexy.

Hypercortisolemia due to Cushing's disease, one of the critical issues in pituitary tumors is associated with significant morbidity and increased mortality. Despite the advances in neurosurgical techniques, patients still face relatively low remission rates and high recurrence rates compared to other pituitary tumors. Pituitary directed therapies for Cushing's disease are currently limited but include the recently approved SRL pasireotide and the dopamine agonist cabergoline. TMZ is reserved for aggressive or malignant cases. Several new therapies and including CDK 2/cyclin E inhibitors, EGFR tyrosine kinase inhibitors, retinoic acid, HSP90 inhibitors, monoclonal ACTH antibodies, TR4 inhibition, and microRNAs are under investigation. These novel avenues of therapy are reviewed by Langlois et al. Fuertes et al. focus on retinoic acid, and Araki and Liu discuss the complex role of cell cycle regulation in Cushing's disease.

## Author Contributions

HF wrote the initial draft. TA and OC edited and corrected the text.

### Conflict of Interest Statement

The authors declare that the research was conducted in the absence of any commercial or financial relationships that could be construed as a potential conflict of interest.

